# Gas evolution in electrochemical flow cell reactors induces resistance gradients with consequences for the positioning of the reference electrode†

**DOI:** 10.1039/d1ra05345k

**Published:** 2021-08-20

**Authors:** Yannick Jännsch, Martin Hämmerle, Jane J. Leung, Elfriede Simon, Maximilian Fleischer, Ralf Moos

**Affiliations:** Department for Functional Materials, University of Bayreuth 95440 Bayreuth Germany functional.materials@uni-bayreuth.de; Siemens Energy Global GmbH & Co. KG Otto-Hahn-Ring 6 81739 Muenchen Germany

## Abstract

With the transfer of the electrochemical CO_2_-reduction from academic labs towards industrial application, one major factor is the increase in current density. This can be achieved *via* the usage of a gas diffusion electrode. It allows for electrochemical reactions at the three-phase boundary between gaseous CO_2_, liquid electrolyte and electrocatalyst. Thus, current densities in commercially relevant magnitudes of 200 mA cm^−2^ and beyond can be reached. However, when increasing the current density one faces a new set of challenges, unknown from low current experiments. Here, we address the issue of gas evolution causing a local increase in resistance and the impact on the operation of flow cells with gas diffusion electrodes. We set up a simple simulation model and compared the results with experiments on a real setup. As a result, the gas evolution's strong impact on current-, potential- and resistance-distributions along the flow axis can be described. Main consequence is that the positioning of the reference electrode has a significant effect on the locally measured IR-drop and thus on the measured or applied potential. Therefore, data from different setups must be compared with great care, especially with respect to the potentials, on which the cell is operated.

## Abstract 2

The gas evolution during electrolysis in flow cells results in inhomogeneous distributions of resistance, current and voltage along the flow axis.

## Graphical abstract

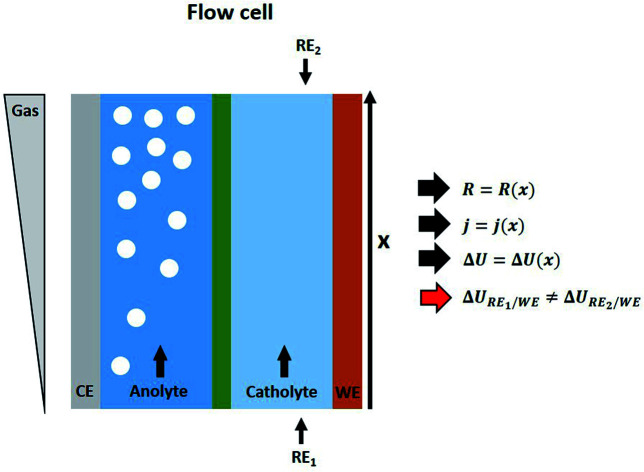

## 1. Introduction

Electrochemical CO_2_ reduction (CO_2_RR) is a promising pathway towards closing the carbon cycle.^1–3^ Flow cells in combination with gas diffusion electrodes (GDEs) are a favourable concept for the scaling up of electrochemical gas conversion under higher current densities. This is particularly valid for the field of electrochemical CO_2_ reduction.^4–7^ Due to the low solubility of CO_2_ in water, a gaseous CO_2_ supply is the most effective way to increase CO_2_ availability on the catalyst surface and thus increase the applicable current density.^8^ Gas diffusion electrodes consist of a microporous, gas permeable electrode substrate that is coated with an electrocatalyst. It divides the electrolyte flow on one side from the gas flow on the other side. The gaseous CO_2_ diffuses through the electrode to the catalyst layer, where the conversion reaction can take place at the three-phase boundary between reactant gas, catalyst, and electrolyte.^9^

**Fig. 1:**
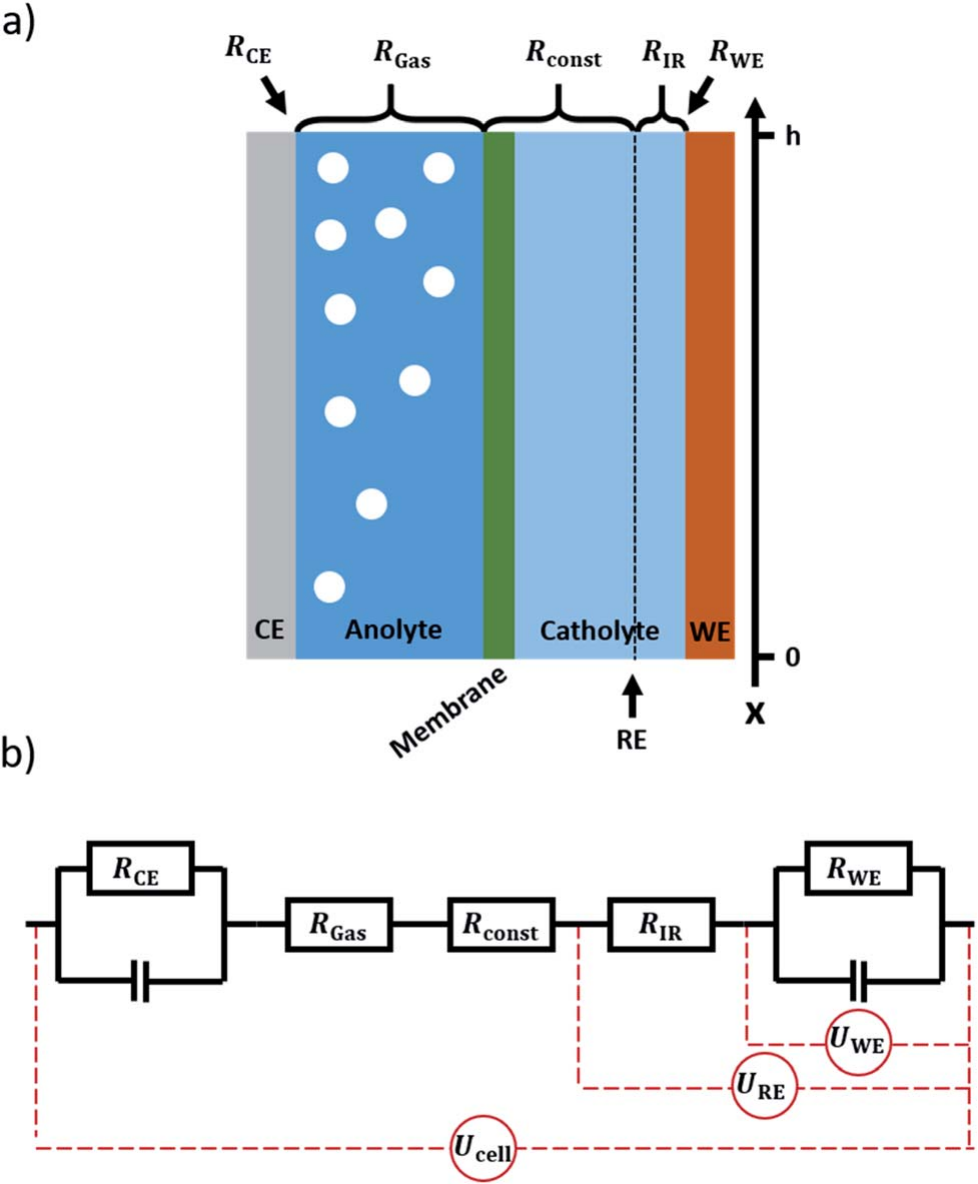
(a) Scheme of the modelled cell. From left to right, it consists of a gas-producing counter-electrode, the anolyte compartment, an ion exchange membrane, the catholyte compartment and a working electrode. Both anolyte and catholyte flow in *x*-direction. The position of the reference electrode in relation to the *x*-axis is marked by a dotted line. It is assumed that gas evolution only occurs at the counter electrode. (b) The corresponding equivalent circuit. Working and counter electrode are represented by an RC-Element. The anolyte is represented by the resistance *R*_Gas_, which changes with gas evolution. Membrane and most of the catholyte are summed up in the unchanging *R*_const_. The resistance of the catholyte slice between working electrode and reference electrode is denoted *R*_IR_. *U*_cell_ denotes the cell voltage applied to the system, *U*_WE_describes the actual potential drop at the working electrode, and *U*_RE_ denotes the potential drop as measured by a reference electrode, hence including the IR-drop.

As the operation of gas diffusion electrodes is challenging, they are commonly only applied at the cathode side of a CO_2_RR flow cell.^10–12^ At the anode side, the oxygen evolution reaction usually occurs at a solid electrode in contact with the electrolyte. As water from the electrolyte is used as the reactant for oxygen evolution, a solid electrode can reach sufficient current densities. While in the case of a gas diffusion electrode, most gaseous products leave the system *via* the gas channel, there is no such option if using a solid electrode. Thus, on the anode side (solid electrode) all the produced oxygen is fed to the electrolyte compartment, forming a dispersion of gas bubbles therein, as depicted in Fig. 1a. With current densities in the magnitude of 100 mA cm^−2^ and above for practical applications,^2,13,14^ the volume of gas produced during electrolysis can have a significant impact on the electrical properties of the system.^15^ As the amount of gas in the electrolyte accumulates along the electrolyte flow axis (henceforth referred to as the *x*-axis, see Fig. 1a), gradients in resistance, voltage and current are expected to arise along this axis. In order to calculate these gradients, a model was set up allowing for the *x*-dependent calculation of the respective properties of the system.

## 2. Theory

In order to express its *x*-dependency, the current *I* in units of A is not suitable. Instead, a one-dimensional current density *j* in units of A cm^−1^ is used. It is defined as follows:1
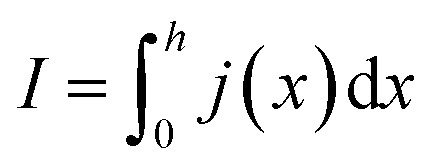
Herein, *h* denotes the total length of the electrode along the *x*-axis and *I* the total current applied. Note that eqn (1) assumes homogeneous distributions along the *y*- and *z*-axis. Even though this may not be entirely the case in reality, the chosen formulation is suited to calculate the desired properties, thus the assumption was made. Similar to the current, the resistance has to be transformed to reflect its *x*-dependency. In this case, a one-dimensional specific resistance *ρ* in units of Ω cm can be defined as:2
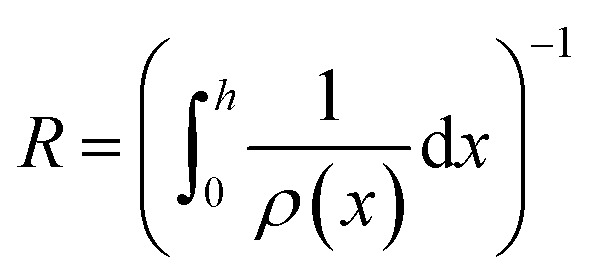
where *R* denotes a resistance over the entire height of the system. The voltage applied to the system (cell voltage) can hence be defined as:3
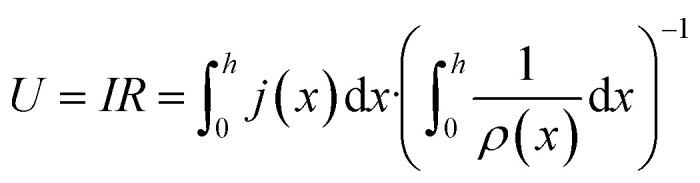
Note that the overall voltage is not *x*-dependent, as the cell voltage is given for the entire system. What can be *x*-dependent, however, are voltage drops Δ*U*′(*x*) over individual resistances *ρ*′(*x*). These can be calculated as:

4 Δ*U*′(*x*) = *j*(*x*)*ρ*′(*x*)

In order to calculate the entities introduced above, the influence of gas evolution on the electrolyte resistance and the behaviour of the charge transfer resistances are formulated in the following.

### 2.1 Gas evolution and electrolyte resistance

The volume of gas that evolves, *V*, can be calculated *via* the faradaic laws of electrolysis and the ideal gas law as follows (details on the derivation can be found in the ESI†):5
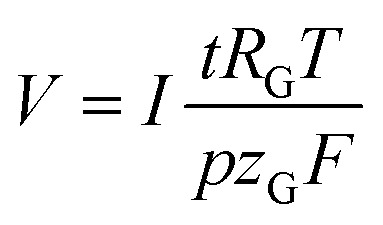
Here, *t* denotes the time passed, *R*_G_ the universal gas constant, *T* the temperature, *p* the pressure, *z*_G_ the number of moles of electrons involved in the formation of one mole of gas molecules, and *F* the Faraday constant. The presence of factor *t* renders this expression time-dependent. It turns out, however, that when expressing the gas evolution as a gas flow, *Q*_G_, the model can be solved stationary:6
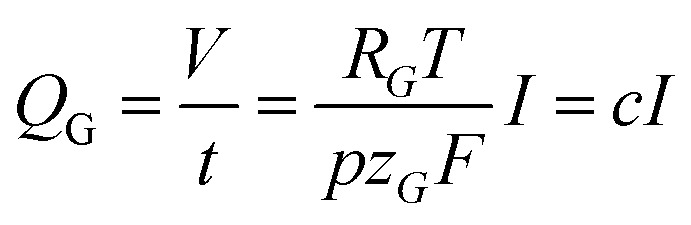
In eqn (6)
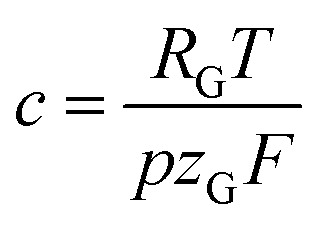
 is a constant summarizing all involved constants. Note, that temperature and pressure most probably show a small *x*-dependence as well. However, the dependencies can in practice be minimized by low cell height, high electrolyte flow rates and active temperature control, and are thus treated as constant.

Finally, this expression can be transformed into an *x*-dependent form using the local current density instead of the total current:7
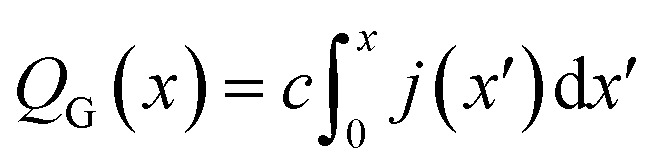


In this way, the cumulative current from the electrolyte inlet at *x*′ = 0 all the way to *x*′ = *x* can also be transformed into a cumulative gas flow.

Next, we consider the evolution of gas as a change in electrolyte resistance, as the electrolyte can be considered as a suspension of non-conducting gas bubbles in the conducting electrolyte medium. The amount of gas in the system can be expressed as the volume fraction *f*:8
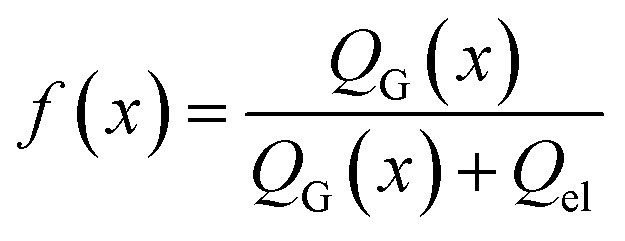
where *Q*_el_ denotes the flow of pure electrolyte, which is an *x*-independent quantity. Various models for the calculation of suspension resistances have been proposed.^16–19^ Even though they were originally developed for electrical resistances, they can be applied to electrolytic resistances as well.^20^ Assuming the suspended component is an insulator, the resistance of the suspension can be calculated as follows, according to the respective models from Maxwell,^16^ Bruggemann,^17^ Tobias,^18^ or Prager:^19^9a
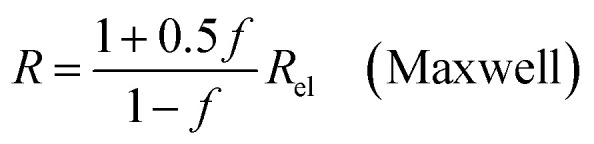
9b
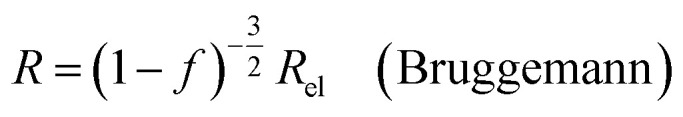
9c
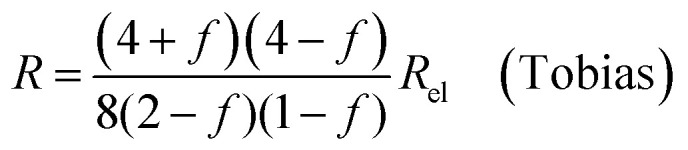
9d
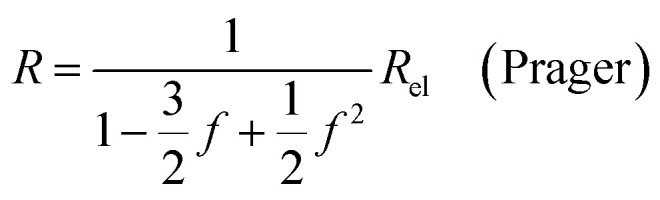


**Fig. 2:**
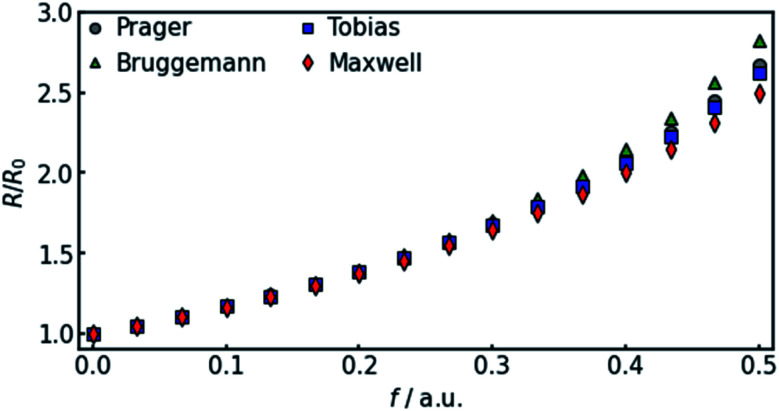
Comparison of the four different equations given in eqn (9a–d) that calculate the resistance of a suspension depending on the volume-fraction of the suspended, isolating component.

In all equations, *R*_el_ denotes the pure electrolytes resistance. All four equations are plotted in Fig. 2. It can be observed that the four equations differ significantly for the description of suspensions with a high fraction of the suspended phase. If the volume fraction *f* stays below 0.3, all four equations result in nearly identical values for the suspension's resistance.

The results shown in the following are calculated using the Maxwell equation (eqn (9a)), as it is the most simple and traditional model. In order to adapt the equation to the use of specific resistance, the resistance can simply be replaced by its specific equivalent:10
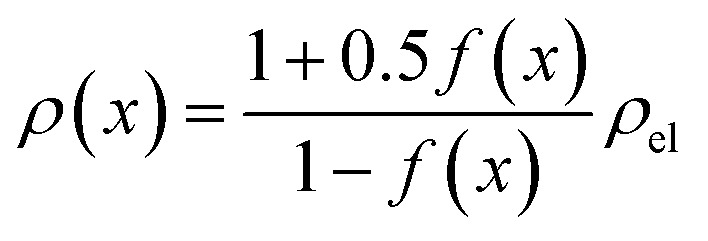


The resistance of the pure electrolyte is most probably not independent of *x*. Ions will be consumed and/or generated in the electrochemical process, changing ion concentrations and charge carrier density along the electrolyte flow.^21^ This results in a series of complex issues within the system.

- The electrolytic conductivity changes. As classical electrolyte conductivity theory (Kohlrausch, Debye–Hückel–Onsager) is not suited for high ion concentrations as commonly used in flow cell reactors, especially if dealing with asymmetrical electrolytes, formalizing this aspect is not trivial.^22,23^

- The concentrations of reactants and products change along the *x*-axis. This will impact potentials according to the Nernst equation. Looking at CO_2_RR in particular, the large number of possible reactions and unclear corresponding pathways make a discrete calculation of the effects very complicated.

- A changing pH will also impact potential measurements using Ag/AgCl reference electrodes.

- Additional complexity is added to the problem by the Wien effect, the relaxation effect and the electrophoretic effect.^24^

In order to keep the complexity of the model to a reasonable level, it was decided not to consider changes in pure electrolyte conductivity. High flow rates for both electrolyte and feed gas were chosen in order to minimize the effects named above. Gradients in the electrolyte concentration have been investigated by Kas *et al.*^25^

### 2.2 Charge transfer resistances

The inhomogeneous electrolyte resistance (as described above) will cause a similarly inhomogeneous distribution of current density. This will in turn cause the charge transfer resistances at the electrodes to change, as they cannot be assumed to be ideal ohmic resistances but obey the Butler–Volmer equation. Starting with the latter, the *x*-dependency of the charge transfer resistances can be derived. It can be simplified to accommodate for one polarity only, as a system under operation with direct current and elevated current density is considered. The simplified Butler–Volmer equation is shown in eqn (11).11
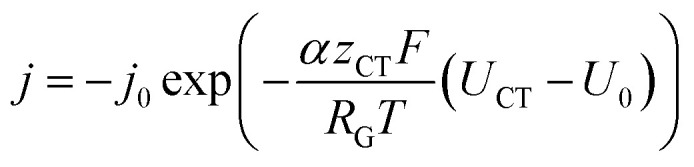
*j*_0_ is the exchange current density, *α* is the charge transfer coefficient, *z*_CT_ is the number of electrons involved, *U*_CT_ is the actual potential, and *U*_0_ is the equilibrium potential. Note that *z*_CT_ and the previously introduced *z*_G_ may differ, as the first describes the number of electrons involved in a single reaction step, whereas the second defines the number of electrons needed to form one molecule of gas. In order to simplify eqn (11), the constant factor 
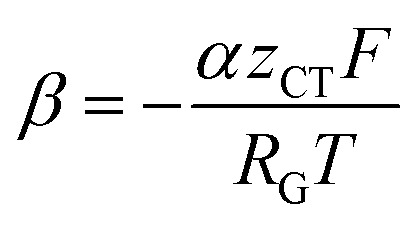
 can be introduced. Also, in order to include the specific resistance of the respective electrode, the voltage *U*_CT_ can be substituted by the *x*-dependent term *U*_CT_(*x*) = *j*(*x*)*ρ*_CT_(*x*), where *ρ*_CT_ denotes the specific charge transfer resistance of the respective electrode. This results in the equation:

12 *j*(*x*) = −*j*_0_ exp(*β*(*j*(*x*)*ρ*_CT_(*x*) − *U*_0_))

By introducing an empirical pair of current density *j*_e_ and specific resistance *ρ*_e_, which are discussed further in Section 3.2, *j*_0_ can, based on eqn (12), be determined as:13
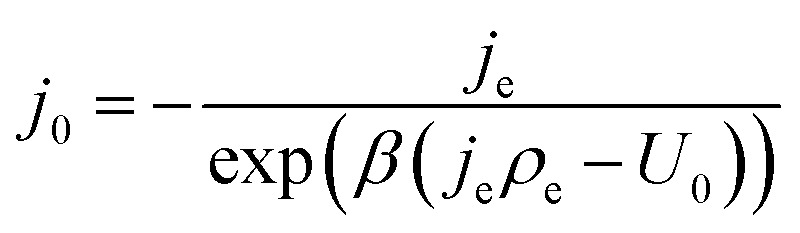
If this expression is inserted back into eqn (12) and solved for *ρ*(*x*) (see ESI† for details), the following expression results:14
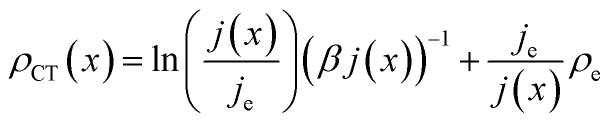


Note that eqn (14) does not rely on *j*_0_ and *U*_0_ anymore. This is of an advantage, as these entities can hardly be estimated. Henceforth, the specific charge transfer resistance *ρ*_CT_ of the anode and cathode are referred to as *ρ*_CE_ and *ρ*_WE_, respectively.

Combining gas evolution, its impact on the electrolyte resistance, and the behavior of the charge transfer resistances, a model can be setup.

## 3. The model

The system to be modelled is depicted in Fig. 1a. It consists of a working electrode and a counter electrode, with their respective electrolyte compartments. The latter are divided by an ion exchange membrane. The corresponding equivalent circuit is shown in Fig. 1b. It consists of a series of resistances, attributed to electrolyte and ion exchange membrane. Working and counter electrode are represented by RC-elements, in order to consider both double layer capacity and charge transfer resistance. As a direct current will be applied to the system, the capacitors for both working and counter electrode will block, thus a series of resistances remains. The counter and working electrode resistances *R*_CE_ and *R*_WE_ are charge transfer resistances. The electrolyte is sliced formally into three parts. Adjacent to the counter electrode, the slice of electrolyte affected by gas evolution at the anode, *R*_Gas_, is situated. It is followed by a constant resistance *R*_const_ to accommodate for the ion exchange membrane and gas-unaffected electrolyte areas. Finally, a small slice of the electrolyte next to the working electrode is expressed as a separate constant resistance *R*_IR_. This resistance represents the electrolyte resistance over the distance between the working electrode and the position where a reference electrode would be inserted. This way, the IR-drop and potential as measured with a reference electrode can be included in the model. As all resistances are connected in series, the overall resistance, expressed as specific resistance, can be defined as

15 *ρ*(*x*) = *ρ*_CE_(*x*) + *ρ*_Gas_(*x*) + *ρ*_const_ + *ρ*_IR_ + *ρ*_WE_(*x*)

### 3.1 Calculation

In order to perform calculations, a discrete vector is defined for every *x*-dependent variable. Using *n* grid points, each grid point represents a slice of the system, orthogonal to the *x*-axis and with a height of:16
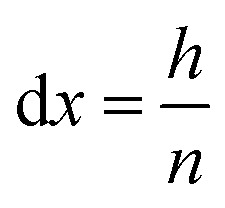


At the initial state of the system, it is assumed that no gas is present in the electrolyte. This results in a gradient free state. The one-dimensional current density can thus be uniformly initiated as:17
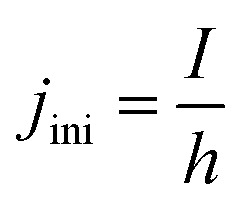


The same is valid for all five partial resistances (eqn (15)) in the system. For any partial resistance *R*_a_, the specific counterpart *ρ*_a_ can be calculated as:

18 *ρ*_a_ = *R*_a_*h*

At this point, an iterative process is conducted:

(1) Calculate gas flow (eqn (7)).

(2) Update specific electrolyte resistance (eqn (8) and (10)).

(3) Update specific charge transfer resistances (omittable in first iteration, eqn (14)).

(4) Calculate overall specific resistance (eqn (15)).

(5) Calculate overall cell voltage (eqn (3)).

(6) Update current distribution using the overall voltage and resistance using the relation19
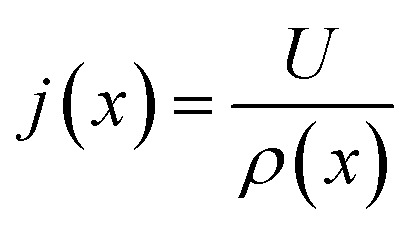


The process is repeated, until it converges to a stationary state.

### 3.2 Initial values

The developed algorithm requires several parameters to conduct the calculation. They were chosen to represent an experimental setup at our labs, built around a Micro Flow Cell from *Electrocell* (electrode height *h* = 3.42 cm, electrode area = 10 cm^2^ additional information in the ESI†). It is most commonly operated galvanostatically with −200 mA cm^−2^ applied current density. Temperature and pressure are controlled to values of 20 °C and 30 mbar above atmosphere, respectively. The electrolyte flow on the anode side was set to 10 L h^−1^.

The five initial resistances representing the system were approximated using experimental data as follows. Fig. S1† shows impedance measurements in both two- and three-electrode setups. In the two-electrode setup when measuring at high frequency, the sum of all three electrolyte resistances *R*_IR_, *R*_const_, and *R*_Gas_ was measured to be 2 Ω. At this point, the contributions of electrolyte at the anode side (anolyte), the ion exchange membrane and the electrolyte at the cathode side must be distinguished from one another. The specific resistance of a Nafion 117 membrane is in the magnitude of 15 Ω cm^−1^.^26^ With a thickness of 187 μm and an area of 10 cm^2^, a negligible resistance of 30 mΩ from the membrane can be estimated. Thus, the 2 Ω can be equally divided into an anolyte and catholyte contribution of 1 Ω each. The anolyte contribution can be attributed to the gas-dependent resistance *R*_Gas_. On the cathode side, the 1 Ω must be subdivided once more into *R*_const_ and *R*_IR_. Impedance measurements in a three-electrode setup at high frequencies yield a resistance of 0.1 Ω, which corresponds to *R*_IR_. Thus, *R*_const_ is 0.9 Ω.

The resistance of the entire system under load can be calculated as the quotient of the applied current (−2 A) and the required cell voltage (−7 V), yielding 3.5 Ω. Subtracting the electrolyte resistance of 2 Ω, the sum of the charge transfer resistances *R*_CE_ and *R*_WE_ remains to be 1.5 Ω. As the individual contributions of counter and working electrode cannot be determined, an assumption had to be made at this point. GDEs usually have high roughness and therefore a large surface area. Hence, it was assumed that the charge transfer resistance of the working electrode is lower than the charge transfer resistance of the counter electrode. Thus, *R*_CE_ was set to 0.8 Ω and *R*_WE_ to 0.7 Ω. It has to be denoted that the measured resistance under load involves gas evolution. Yet, it is used as the initial resistance at the start of the iteration process, where no gas evolution is assumed. This results in a slight overestimation of the resistance of the system. Fig. S2† shows that this results in a small quantitative error, but does not impact the qualitative behavior.

Converting current and resistances to current density and specific resistances (eqn (1) and (2)), *ρ*_CE,ini_, *ρ*_WE,ini_ and *j*_ini_ also served as empirical values *ρ*_CE,e_, *ρ*_WE,e_, and *j*_e_ for the calculation of charge transfer resistances, respectively.

Further parameters are the number of involved electrons *z*_CT_ and the charge transfer coefficient *α*. Taking a look at mechanisms in electrochemistry, the individual steps usually take only 1 or 2 electrons. Thus, *z*_CT_ was set to 1 and *α* was set to 0.5.

In order to calculate the gas evolution, the number of electrons per evolving gas molecule *z*_G_ was set to 4 as oxygen evolution is assumed.

All initial values are summarized in Table 1.

**Table 1:** Initial values for the first run of the simulation

Parameter	Value
*T*	298.15 K
*p*	1043 mbar
*Q*_el_	10 L h^−1^
*z*_G_	4
*z*_CT_	1
*α*	0.5
*I*	−2 A
*R*_CE_ a	0.8 Ω
*R*_Gas_ a	1.0 Ω
*R*_const_	0.9 Ω
*R*_IR_	0.1 Ω
*R*_WE_ a	0.7 Ω

a These values change during the iteration process.

## 4. Results

### 4.1 Convergence

**Fig. 3:**
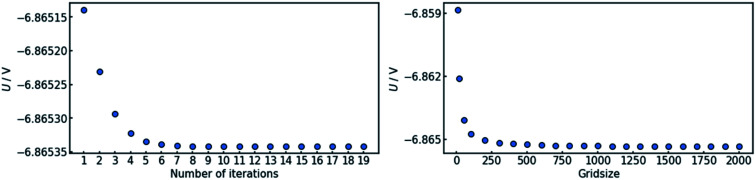
Convergence behavior of the algorithm for (a) the number of iterations and (b) the grid size. In both cases, the data converges within the tested interval. The criterion chosen for convergence is the stability of the cell voltage U.

In order to assure the accuracy of the calculation, both the number of iteration steps and the number of grid points along the *x*-axis must be sufficiently large. This was ensured by carrying out a parameter sweep for both entities (Fig. 3). The cell voltage was used as the indicator for conversion. It is suited for the purpose, as it is affected by changes all along the *x*-axis and it is a single value representing the entire state of the cell.

The sweep of the number of iteration steps was conducted under use of the maximum tested value for grid size and *vice versa*. It can be deduced that a number of 20 iteration steps with a grid size of 2000 points is sufficient in order to reach an acceptable level of convergence.

Fig. 3 also shows that a steady state in terms of cell voltage is reached. Hence, all results calculated herein for galvanostatic operation are also valid for potentiostatic operation.

### 4.2 *x*-Dependency of resistance and current

**Fig. 4:**
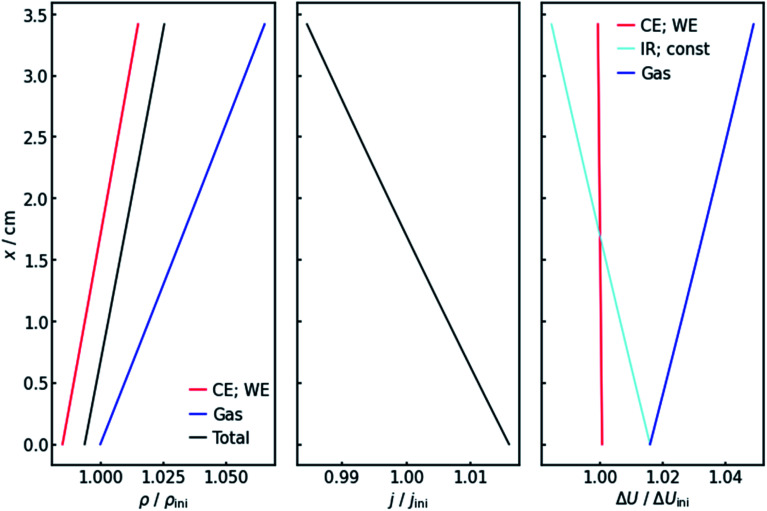
Values of the specific resistances (left), current density (middle) and voltage drops (right) along the flow axis in the equilibrium state when applying −200 mA cm^−2^. All values are normalized by their initial value without gas evolution at the start of the iteration. Note that the values for the working and counter electrode in the left and right panel are congruent. The same is the case for the voltage drops over the resistances *R*_const_ and *R*_IR_ in the right panel.

Fig. 4 (left panel) shows the resulting specific resistance distributions, each normalized by their initial value. The resistance of the gas-affected anolyte *ρ*_Gas_ stays the same at the electrolyte inlet. This makes sense as the amount of gas in the stream should be zero at the electrolyte inlet. With increasing height and thus gas accumulation, however, the resistance increases as expected. The charge transfer resistances show a similar trend (Fig. 4, left panel), but with the important difference that they drop below their initial value at the electrolyte inlet. This can be explained by the current distribution, also shown in Fig. 4 (middle panel). Due to the resistance profile, larger currents tend to flow towards the electrolyte entrance. Because of the Butler–Volmer relation, if the current increases, the charge-transfer resistance at the respective height on the *x*-axis has to decrease. Another consequence of the inhomogeneous current distribution is that the resistance does not show a linear *x*-dependency but has a slight decrease in (absolute) slope. As a consequence of the above, the cumulative cell resistance (Fig. 4) shows a near-linear positive *x*-dependency starting below the initial value.

### 4.3 Voltages

Of course, the voltage is affected by the gradients in resistance and current. The overall cell voltage of the system decreases from −6.800 V to −6.866 V, as the gas evolution increases the overall resistance. For the voltage drop Δ*U* over the gas-containing anolyte, a rise along the *x*-axis can be observed as expected (Fig. 4, right panel). Looking at the voltage drop at the working electrode, a slight *x*-dependency can be observed. However, even though the inhomogeneities in current and resistance are in the magnitude of 3 to 6%, the voltage drop at the WE only changes by around 0.1%. This is due to the nature of the charge-transfer resistances. As current increases, the resistance decreases, keeping the effective voltage drop nearly constant.

**Fig. 5:**
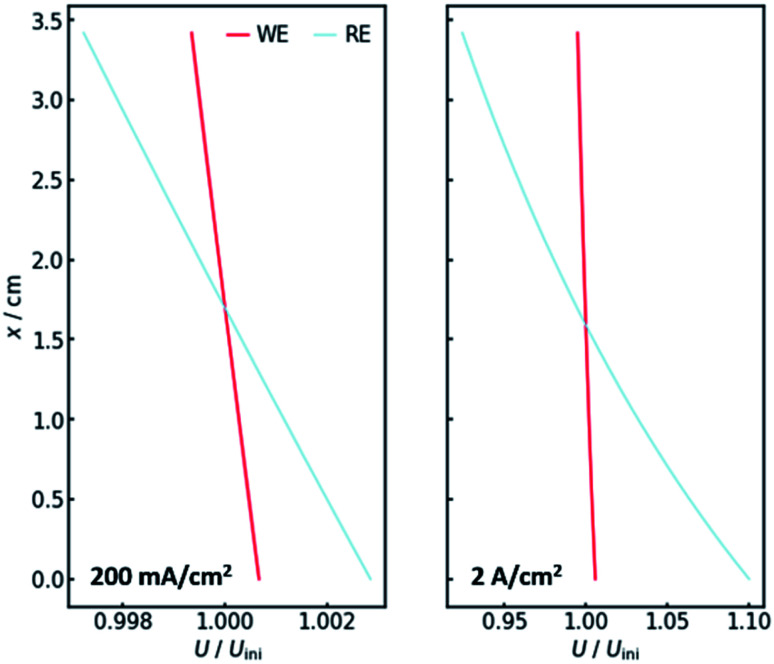
The voltage drop at the working electrode (WE, red) and the potential as measured at the reference electrode position (RE, blue) for current densities of 200 mA cm^−2^ (left) and 2 A cm^−2^ (right) along the *x*-axis. All values are normalized by their value without gas evolution at the start of the iteration.

The voltage drop over the electrolyte slice that causes the IR-drop, however, has a steeper gradient, as a result of the current distribution and the constant specific resistance *ρ*_IR_. This affects measurements using a reference electrode. The actual potential drop at the working electrode (Fig. 5) is nearly constant. However, if measuring the potential difference between the working and a reference electrode, the gradient is significantly steeper. As Fig. 5 shows, the effect is stronger for higher current densities. Fig. 5 also shows that the *ideal* insertion height of the reference electrode, meaning the height at which measured potential and working electrode potential drop are equal, shifts towards the electrolyte entry point with increasing current density.

Two conclusions can be drawn at this point. On the one hand, the potential drop at the working electrode is nearly constant along the *x*-axis, thus there needs to be no concern with different reactions taking place along the axis. On the other hand, a significant gradient in the measured potential can be observed at the position of the reference electrode. As a consequence, the positioning of the reference along the *x*-axis in real flow cell reactors has a significant impact on the IR-drop and thus the measured potential, even though the distance between working and reference electrode may not be altered. This is especially valid for high current densities, when much oxygen evolves and many bubbles are formed.

### 4.4 Experimental validation

**Fig. 6:**
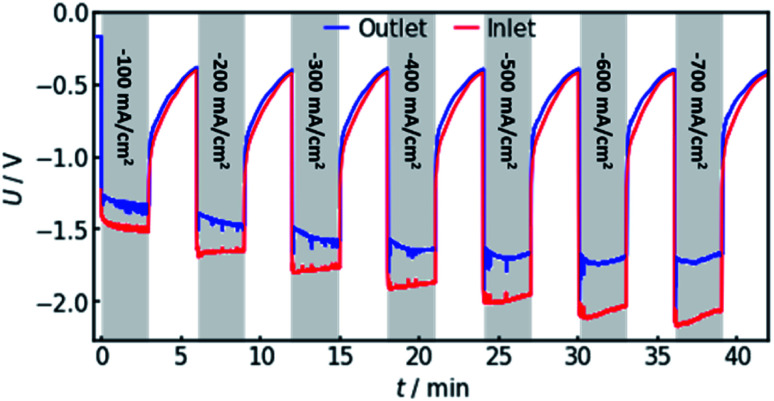
Experimental measurement of the voltages at both the electrolyte inlet and outlet, using currents from −100 to −700 mA cm^−2^. The red line represents the potential as measured by an Ag/AgCl reference electrode in the electrolyte inlet. A similar, simultaneous measurement at the electrolyte outlet is represented by the blue line. The voltage offset was determined as the difference of average measured potential at the two electrodes for each applied current density.

In order to confirm the theoretical results, measurements on a real flow cell reactor were conducted. Therefore, two reference electrodes were installed, one at the electrolyte inlet and one at the electrolyte outlet. While applying a constant current, the potential as measured with both electrodes was observed and compared. This was done for current densities from −100 to −700 mA cm^−2^. The results are shown in Fig. 6.

**Fig. 7:**
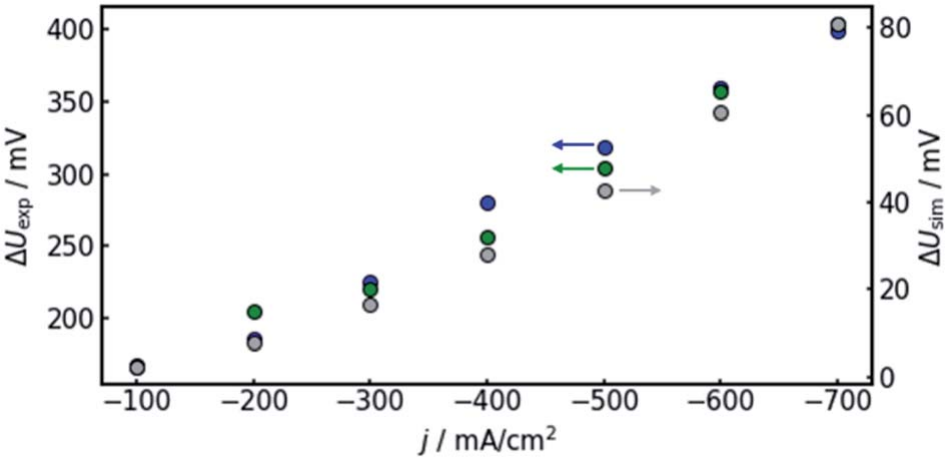
Comparison of the voltage offset between the electrolyte inlet and outlet, as predicted by the model (grey, right axis) and the corresponding experimental values, determined in two separate runs (green and blue, left axis) at various current densities.

It can be observed that the potential measurements at the two positions differ in the magnitude of 100 mV under load, while the potential difference was measured to be smaller than 10 mV when in open circuit state. Fig. 7 shows the potential difference in dependence of the applied current density. Calculations with the same current densities were conducted, also comparing the potential at the reference electrode position at the electrolyte inlet and outlet, respectively.

The results show that the simulation predicts an increasing difference in measured potentials with increasing current density, but underestimates the effect in two ways. First, the slope of the simulated curve is only one third as steep as the respective experimental curve. This can have various reasons. Choosing the Maxwell equation, the least drastic function (Fig. 2) was used in order to quantify how the amount of gas changes the resistance of the electrolyte/gas mixture. Additionally, the quantitative behavior is strongly influenced by the initial parameters, which, as described above in Section 3.2, bear a certain uncertainty. Lastly, the electrolyte effects mentioned in Section 2.1 are not considered by the calculation.

The second difference between simulation and experimental data is that the latter appears to have an offset from the former. Extrapolating the given data to 0 A, one would assume the difference between measured potential at the electrolyte inlet and outlet would approach zero as well. While approximately 10 mV of difference can be attributed to variance between the two reference electrodes used in the experiment, the data shown in Fig. 7 suggest a value larger than 100 mV. The model, in turn, is mathematically bound to approach equal voltages at inlet and outlet if the current approaches 0 A. The discrepancy could again be due to electrolyte effects or measurement inaccuracies due to gas bubbles near the reference electrode in the electrolyte outlet.

Due to the reasons discussed in the previous paragraphs and in Section 2.1, a poor quantitative match between experiment and model is not surprising. Nevertheless, a qualitative agreement exists, as the general trend of the simulation can be confirmed by the experiment. Thus, it can be deduced that gas evolution leads to IR-like errors in the potential measurement with the magnitude of the error varying along the *x*-axis. In consequence, the positioning of the reference electrode strongly influences the measured potential. Thus, two seemingly identical experiments can yield very different results, if the reference is installed at different positions. This may also form part of the explanation as to why comparability remains an issue in the field of CO_2_-reduction.^27^

## 5. Conclusion

All in all, an analytical model was constructed that calculates the gradients of current, resistance and voltage along the flow axis of an electrochemical flow cell due to gas evolution. As expected, due to higher amounts of gas in the electrolyte near the electrolyte outlet, the resistance rises along the flow axis. This results in an inverse current distribution, meaning higher currents occur near the electrolyte inlet and lower currents occur at the electrolyte outlet. The resulting gradient of the voltage drop at the working electrode is negligible. This is important, as the voltage drop at an electrochemical catalyst has a large impact on its product distribution. The gradient being small induces the absence of significant differences in the reaction at the catalyst along the flow axis.

A larger gradient can be attributed to the IR-drop, especially at high currents. In consequence, the measured potential using a reference electrode strongly depends on the installation point of the reference electrode along the flow axis. This proposes consequences for the conduction of potentiostatic experiments, as well as for precise voltage measurements in galvanostatic experiments. As flowcell geometry, operation parameters and the installation point of the reference electrode differ between different research groups, they may find that despite operating the same catalyst at supposedly the same potential, current densities and product distribution may differ. In order to approximate the magnitude of the error, an experiment as depicted in Fig. 6 could be a sensible test to conduct for each setup and the corresponding operational parameters.

The presented algorithm does not accommodate for all gradient effects in a flow cell. Concentration changes in the electrolyte and feed gas are likely to have additional effects. Experimental data showing a stronger *x*-dependence than the simulation supports this hypothesis. The incorporation of such effects into the given model exceeds the scope of this study but should be considered in future work.

## Funding

This work was supported by the Bavarian Research Foundation (BFS) [grant number AZ-1391-19]. This publication was funded by the German Research Foundation (DFG) and the University of Bayreuth in the funding programme Open Access Publishing. The authors greatly acknowledge the funding.

## Author contributions

Yannick Jännsch: conceptualization, investigation, methodology, writing – original draft, writing – review & editing. Martin Hämmerle: supervision, writing – review & editing, funding acquisition. Jane J. Leung: writing – review & editing. Elfriede Simon: writing – review & editing. Maximilian Fleischer: writing – review & editing. Ralf Moos: supervision, writing – review & editing, funding acquisition.

## Conflicts of interest

The authors declare that they have no known conflicting financial interests or personal relationships that could have appeared to influence the work reported in this paper.

## Supplementary Material

RA-011-D1RA05345K-s001
