# Supplementary material for: Gas evolution in electrochemical flow cell reactors induces resistance gradients with consequences for the positioning of the reference electrode
Source: RSC Adv. 2021 Aug 20;11(45):28189–97. doi: 10.1039/d1ra05345k (PMC9038112; doi:10.1039/d1ra05345k)
Supplement: RA-011-D1RA05345K-s001 [file RA-011-D1RA05345K-s001.pdf]

Electronic Supplementary Material (ESI) for RSC Advances.
This journal is © The Royal Society of Chemistry 2021

Supporting Information for

Gas evolution in electrochemical flow cell reactors induces resistance gradients with consequences for the positioning of the reference electrode.

Yannick Jännsch[a,*], Martin Hämmerle[a], Jane J. Leung[b], Elfriede Simon[b], Maximilian Fleischer[b], Ralf Moos[a]

[a] Department for Functional Materials, University of Bayreuth, 95440 Bayreuth, Germany

[b] Siemens Energy Global GmbH & Co. KG, Otto-Hahn-Ring 6, 81739 Muenchen, Germany

* Corresponding author. E-mail address: functional.materials@uni-bayreuth.de

1. Derivation of Equation 5

Equation 5 is based on the ideal gas law:

$$V = \frac{nR_G T}{p}$$

According to the Faradaic laws, the following is valid as well:

$$It = nz_G F$$

The latter can be rewritten as

$$n = \frac{It}{z_G F}$$

If inserting the resulting expression into the ideal gas law, one recieves Equation 5:

$$V = \frac{ItR_G T}{pz_G F}$$

2. Derivation of Equation 14

Equation 12, in the article was defined as:

$$j(x) = -j_0 \cdot \exp\big(\beta(j(x)\rho_{\mathrm{CT}}(x) - U_0)\big)$$

In order to get to equation 14 the following expression (Equation 13) had to be inserted for j_0:

$$j_0 = -\frac{j_{\mathrm{e}}}{\exp\big(\beta(j_{\mathrm{e}}\rho_{\mathrm{e}} - U_0)\big)}$$

The following is a step-by-step solution:

$$\begin{aligned}
j(x) &= -j_0 \cdot \exp\big(\beta(j(x)\rho_{\mathrm{CT}}(x) - U_0)\big)\\
\ln\big(j(x)\big) &= \ln(-j_0) + \beta(j(x)\rho_{CT}(x) - U_0)\\
\ln\big(j(x)\big) &= \ln\left(\frac{j_{\mathrm{e}}}{\exp\big(\beta(j_{\mathrm{e}}\rho_{\mathrm{e}} - U_0)\big)}\right) + \beta j(x)\rho_{CT}(x) - \beta U_0\\
\ln\big(j(x)\big) &= \ln(j_e) - \beta(j_e\rho_e - U_0) + \beta j(x)\rho_{CT}(x) - \beta U_0\\
\ln\big(j(x)\big) &= \ln(j_e) - \beta j_e\rho_e + \beta U_0 + \beta j(x)\rho_{CT}(x) - \beta U_0\\
\beta j(x)\rho_{CT}(x) &= \ln\big(j(x)\big) - \ln(j_e) + \beta j_e\rho_e
\end{aligned}$$

$$\beta j(x)\rho_{CT}(x) = \ln\left(\frac{j(x)}{j_e}\right) + \beta j_e \rho_e$$

$$\rho_{CT}(x) = \ln\left(\frac{j(x)}{j_e}\right) \cdot \left(\beta j(x)\right)^{-1} + \frac{j_e}{j(x)}\,\rho_e$$

3 Experimental

3.1 GDE Preparation

An ink was prepared as a mixture of a Nafion dispersion (20%, Sigma Aldrich) and copper particles (40 - 60 nm, Sigma Aldrich) in the ratio 1:10 in isopropanol. The ink was sonicated for 30 minutes and then dropcasted onto a gas diffusion layer (Freudenberg H23C2) to achieve a catalyst film with a theoretical copper loading of 1,26 mg/cm^2. The GDE was then dried under nitrogen atmosphere.

3.2 Electrochemical Measurements

The GDE was installed in a flow cell (Micro Flow Cell, Electrocell), which also contained a Nafion membrane (N117, Ion Power GmbH) to separate the anolyte from the catholyte channel. The gas channel was supplied with 100 mL/min CO2 (Rießner Gase, grade 5.5). A 1 M $KHCO_3$ solution was used as electrolyte. Both anolyte and catholyte channel were supplied by magnet coupled rotary pumps (M1, March Pumpen GmbH & Co. KG) from the same reservoir at a rate of 10 L/h each. The reservoir was set to a temperature of 20 °C by active cooling with a cryostat (CF41, Julabo). A potentiostat (Autolab PGSTAT302N, Metrohm) with a 10 A Booster (Booster10A, Metrohm) was used to conduct the experiments. Ag/AgCl Reference electrodes in a 3M KCl solution (Metrohm) were used as reference electrodes.

4. Impedance Data

Two-electrode-setup Three-electrode-setup

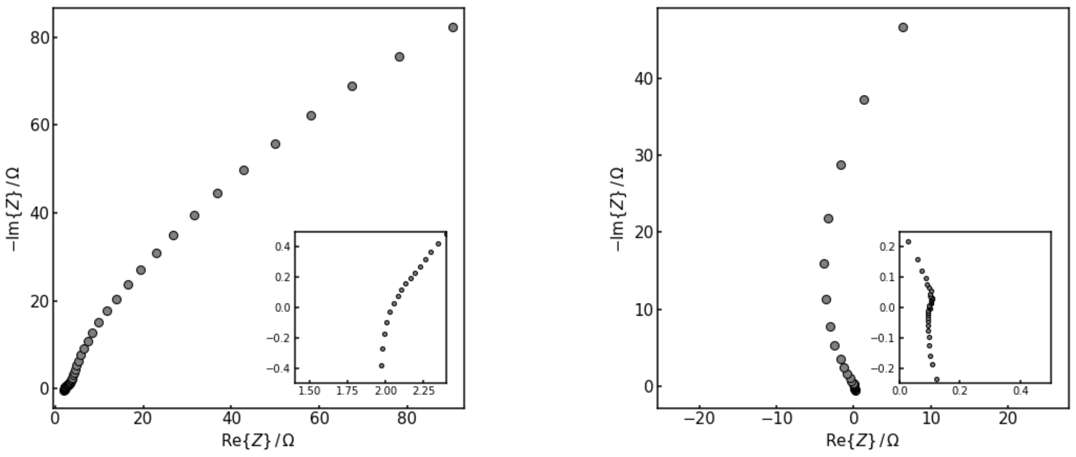

Figure S1: Impedance measurements in two-electrode-setup (left) and three-electrode-setup (right). AC-resistances were determined as the intercept of the real axis.

5. Variation of Counter Electrode Specific Resistance

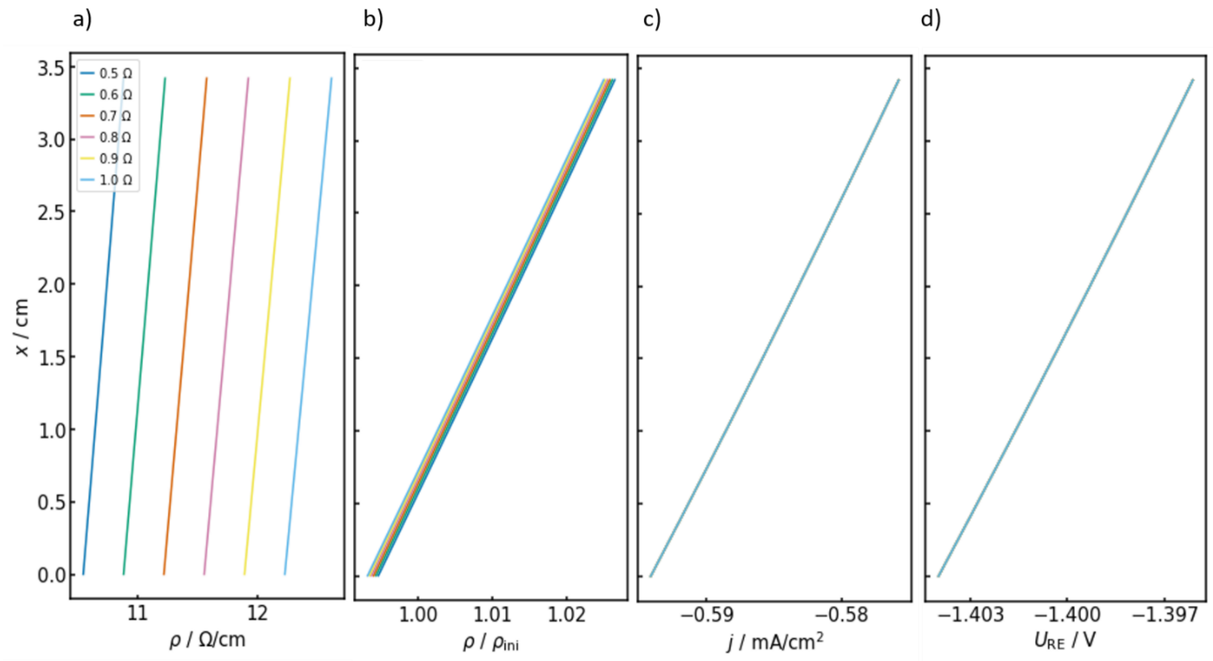

Figure S2: Resulting values for absolute and normalized total specific resistance (a and b respectively), current density (c), and potential as measured by the reference electrode (d) for different values of the resistance of the counter electrode. Panel (a) shows that the total resistance increases with the resistance of the counter electrode as expected. However, if normalizing it by each respective initial value, only minor changes remain. For current and potential, the differences are so small that all lines are congruent. It can be concluded that a slight quantitative inaccuracy when choosing the charge transfer resistances only has a small impact on quantitative results. Qualitatively the behavior of the system is unchanged.

6. Code

In the following, the used code to carry out the simulation is dislosed. Note that for the sake of readability all data-handling functionality has been excluded. Also note, that the *ylib* package is self-written and not officially availlable. The *dotdict* is a simple subclass of the built-in dictionary allowing for dot-notation. It could be replaced with a standard dictionary.

```python
import numpy as np
import scipy.integrate as spintegrate
from ylib import dotdict

###--------------------Initialize------------
def gen_parameters(**kwargs):
    parameter=dotdict({
        "n_ch":200,
        "steps":20,
        "I":-2,
        "h":3.42,
        "R_el":1,
        "R_ir":0.1,
        "R_const":0.9,
        "Re_ce":0.8,
        "Re_we":0.6,
        "Ie":-2,
        "T":293.15,
        "p":101325+30e2,
        "R":8.31446261815324,
```

```
        "F":9.64853321233100184e4,
        "alpha":0.5,
        "zCT":1,
        "zGAS":4,
        "Qel":10e-3/3600})
    parameter["beta"]=calc_beta(parameter.alpha, parameter.zCT, parameter.F,
                                 parameter.R, parameter.T)
    parameter["cgas"]=calc_cgas(parameter.R, parameter.T, parameter.p,
                                parameter.zGAS, parameter.F)
    for i in kwargs:
        parameter[i]=kwargs[i]

    return parameter


###-----------------Equations------------------
def integrate(dx,vec):
    return spintegrate.trapz(y=vec,dx=dx)

def inv_integrate(dx,vec):
    vec=1/vec
    return 1/integrate(dx, vec)

def calc_beta(alpha,zCT,F,R,T):
    return -alpha*zCT*F/R/T

def calc_cgas(R,T,p,zGAS,F):
    return(R*T/p/zGAS/F)

def calc_j(U,r):
    return(U/r)

def calc_U(dx,j,r):
    I=integrate(dx, j)
    R=inv_integrate(dx, r)
    return(R*I)

def calc_rct(je,re,beta,j):
    return np.log(j/je)/beta/j+je*re/j

def calc_r1(rel,f):
    return((1+0.5*f)/(1-f)*rel)

def calc_f(q,Qel):
    return(q/(q+Qel))

def calc_q(cgas,j,dx):
    integral=spintegrate.cumtrapz(np.abs(j),dx=dx)
    integral=np.append(np.array([0]),integral)
    integral*=cgas
    return integral


#--------------------Iteration----------------------
def iterate(parameter,dic=True):
    results=gen_results(parameter.n_ch)

    dx=parameter.h/parameter.n_ch

    j=np.array([parameter.I/parameter.h]*parameter.n_ch)

    je=parameter.Ie/parameter.h

    re_ce=parameter.Re_ce*parameter.h
    r_el=parameter.R_el*parameter.h
    r_const=parameter.R_const*parameter.h
    r_ir=parameter.R_ir*parameter.h
```

```
    re_we=parameter.Re_we*parameter.h

    q=np.zeros_like(j)

    for step in range(0,parameter.steps):
        print("step: {s:.0f}".format(s=step),end="\r")
        f=calc_f(q,parameter.Qel)
        r_1=calc_r1(r_el, f)
        r_we=calc_rct(je, re_we, parameter.beta, j)
        r_ce=calc_rct(je, re_ce, parameter.beta, j)
        r=r_ce+r_1+r_const+r_ir+r_we
        U=calc_U(dx,j,r)
               j=calc_j(U,r)
               q=calc_q(parameter.cgas,j,dx)
```
